# Integrative Analysis of Differently Expressed Genes Reveals a 17-Gene Prognosis Signature for Endometrial Carcinoma

**DOI:** 10.1155/2021/4804694

**Published:** 2021-07-14

**Authors:** Anna Wang, Hongyan Guo, Zaiqiu Long

**Affiliations:** ^1^Department of Gynecology, Cancer Hospital of China Medical University, Liaoning Cancer Hospital & Institute, Liaoning, China; ^2^Department of Information Engineering, Shenyang Polytechnic College, Liaoning, China

## Abstract

Endometrial carcinoma (EC) is the fifth widely occurring malignant neoplasm among women all over the world. However, there is still lacking efficacy indicators for EC's prognosis. Here, we analyzed two databases including an RNA-sequencing-based TCGA dataset and a microarray-based GSE106191. After normalizing the raw data, we identified 114 common genes with upregulation and 308 common genes with downregulation in both the TCGA and GSE106191 databases. Bioinformatics analysis showed that the differently expressed genes in EC were related to the IL17 signaling pathway, PI3K-Akt signaling pathway, and cGMP-PKG signaling pathway. Furthermore, we performed the least absolute shrinkage and selection operator (LASSO) Cox regression analysis and generated a signature featuring 17 prognosis-related genes (MAL2, ANKRD22, METTL7B, IL32, ERFE, OAS1, TRPC1, SRPX, RAPGEF4, PSD3, SIMC1, TRPC6, WFS1, PGR, PAMR1, KCNK6, and FAM189A2) and found that it could predict OS in EC patients. The further analysis showed that OAS1, MAL2, ANKRD22, METTL7B, and IL32 were significantly upregulated in EC samples after comparison with normal samples. However, TRPC1, SRPX, RAPGEF4, PSD3, SIMC1, TRPC6, WFS1, PGR, PAMR1, KCNK6, and FAM189A2 were significantly downregulated in EC samples in comparison with normal samples. And correlation analysis showed that our results showed that the expressions of 17 prognosis-related hub genes were significantly correlated based on Pearson correlation. We here offer a newly genetic biomarker for the prediction of EC patients' prognosis.

## 1. Introduction

Endometrial carcinoma (EC) is the fifth commonly occurring malignant neoplasm among women all over the world, with an estimated 382,000 new EC cases and nearly 90,000 deaths in 2018 [1, 2]. Especially in the United States, it is speculated that the number of newly diagnosed ECs will be increasing over time. It is estimated that the occurrence rate is still rising with increasing risk factors for certain ECs, including obesity rate and the aging of the US population [3]. The incidence rate of EC increases after the age of 30, and the peak incidence is within 60 to 69 years. 20% to 30% of patients with EC are diagnosed in the advanced stage during surgery. In the clinic, the five-year survival rate of patients in stage III ranged from 40% to 70% and in stage IV was within 0 to 10% [1, 4]. Despite therapeutic advances having been made, high recurrence rate and metastasis remain to be big challenges [4]. To determine effective therapeutic strategies in ameliorating the prognostic status of EC patients is thus essential.

Presently, as per the World Health Organization (WHO) classification system classification, EC comprises two sorts on the basis of histological features [5]. Endometrioid adenocarcinoma or well-differentiated endometrioid subtypes accounted for 80% over EC cases and was considered as estrogen-dependent type I of EC [1, 4, 6]. Approximately 10% of EC cases were type II, manifested as nonendometrial or poorly differentiated EC. Difference existed in the molecular changes of the two EC types [1, 4, 7]. In general, in the activated oncogene and inactivated tumor suppressor gene, defective DNA repair contributed mainly to the occurrence of neoplasms [1, 8]. For instance, the inactivated tumor suppressor gene *PTEN* accompanied by DNA mismatch repair gene defects manifested as the microsatellite instability phenotype, or activated KRAS2 and/or adhesion molecules genes were detected in the early stage of type I EC [9]. Previous studies have shown that mutated TP53 and Her-2 occurred in type II EC, which was probably caused by the background of the atrophic endometrium. It seems that these molecular changes were specific in type I and type II of ECs [10, 11]. Although many efforts have been made to set up a molecularly based histological classification, it is still urgently needed to identify the gene expression profiles between different histological types of ECs that distinguish normal cells from cancer cells. There have been public reports showing that the differentially expressed genes existed in different histological sorts of EC [12, 13]. However, a limited set of genes were reported in these studies. More and more researches are thus needed to characterize EC and unearth the genes functioning importantly in the mechanisms of EC.

Herein, we attempted to systematically screen more novel differentially expressed genes and related molecular pathways and the clinical significance of the identified genes in EC. To sum up, identification of EC-related genes and gene pathways as well as the clinical implication is conducive to understanding the pathophysiology of this cancer and uncovering the potential diagnostic biomarkers of EC.

## 2. Materials and Methods

### 2.1. Establishment and Verification of Gene Prognostic Model

We carried out the LASSO Cox regression model (R package “glmnet”) to select the candidate genes and subsequently established the prognostic model [14, 15]. We ultimately retained 17 genes and their coefficients and utilized the minimum criteria to determine the penalty parameter (*λ*). We calculated the risk score after centralizing and standardizing (applying the “scale” function in R) the TCGA expression data. The risk score formula was shown as follows: risk score = ∑7i*X*i × *Y*i (*X*: coefficients, *Y*: gene expression level).

### 2.2. Identification of Differentially Expressed Genes (DEGs)

We obtained expression matrixes and platform information from Gene Expression Omnibus (GEO) datasets. The dataset GSE106191 was used, which includes the primary tumor of 66 endometrial cancer patients (64 carcinoma samples and 33 hyperplasia samples). Then, Software R (version 3.5.1, https://www.r-project.org) and “*limma*” packages (http://www.bioconductor.org/) [16] were applied to select the DEGs existing in the EC samples and control samples. These selection criteria were adjusted *p* value < 0.05 and fold change (FC) ≥ 2 or fold change (FC) ≤ 0.5.

### 2.3. Functional Enrichment Analysis

We usually applied Gene Ontology (GO) functional enrichment analysis to describe gene functions, consisting of molecular function (MF), biological process (BP), or cellular component (CC) [17, 18]. And we then utilized the Kyoto Encyclopedia of Genes and Genomes (KEGG) pathway enrichment analysis to identify molecular interaction and relation networks. *p* value < 0.05 was thought to be statistically significant. Based on the DEGs in the GEO datasets, we conducted GO and KEGG enrichment analyses by the online tool DAVID (https://david. http://ncifcrf.gov/home.jsp) [19, 20]. The top significantly enriched analysis results were shown.

### 2.4. Survival Analysis

For identification of prognosis-predicting gens, we integrated the clinical data of EC patients in The Cancer Genome Atlas (TCGA) and carried out Kaplan-Meier curve analysis [21, 22]. The survival curves of DEGs were drew by “survival” package in R. *p* value < 0.05 was denoted as a significantly statistical difference.

### 2.5. Statistical Analysis

SPSS 22.0 software (Chicago, USA) was employed to analyze the data. All representative data were shown as the mean ± standard deviation (SD) [23–25]. We carried out the Students' *t*-test and one-way ANOVA to separately determine the difference existing in two groups and multiple groups. *p* value < 0.05 was denoted as a significantly statistical difference [26–28]. All experiments were performed in three independent times in replicates at one time.

## 3. Results

### 3.1. Screening of DEGs in EC

**Figure 1:**
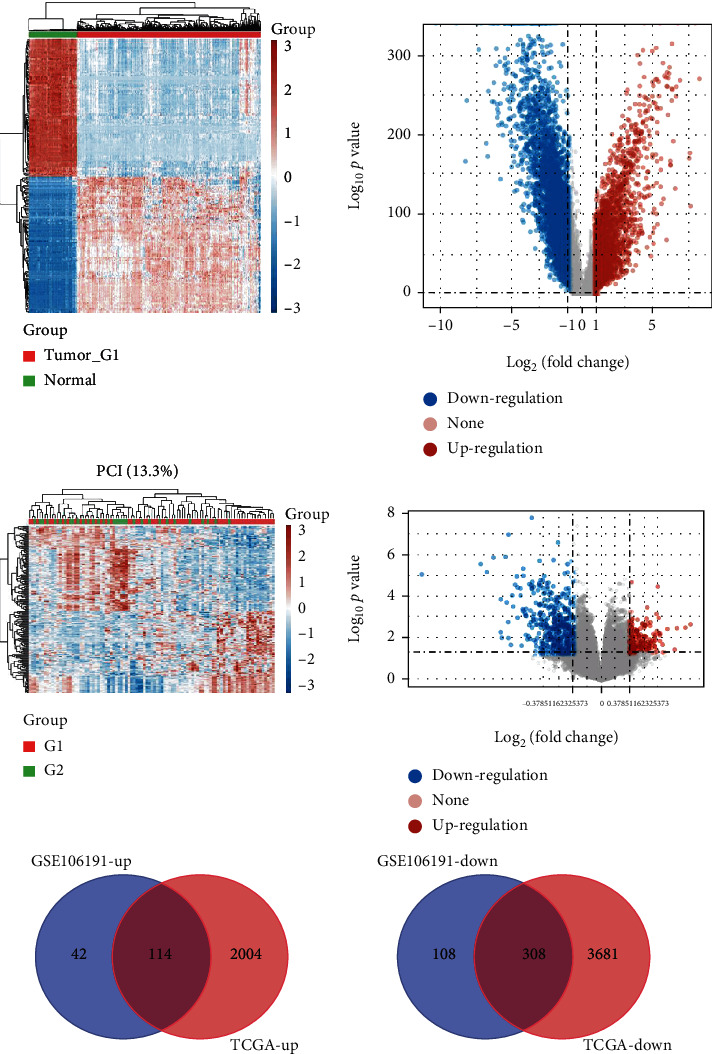
Screening of DEGs in EC. (a, b) Heat map (a) and volcano map (b) identified 2118 genes with upregulation and 3989 genes with downregulation by analyzing the TCGA database. (c, d) Heat map (c) and volcano map (d) showed 156 genes with upregulation and 416 genes with downregulation by analyzing the GSE106191 database. (e, f) Venn map analysis of common upregulated and downregulated genes in EC by analyzing the TCGA and GSE106191 databases.

To identify the DEGs in EC, we analyzed two databases including the RNA-sequencing-based TCGA dataset and microarray-based GSE106191. After normalizing the raw data, we identified 2118 genes with upregulation and 3989 genes with downregulation by analyzing the TCGA database (Figures 1(a) and 1(b)). Meanwhile, we found 156 genes with upregulation and 416 genes with downregulation by analyzing the GSE106191 database (Figures 1(c) and 1(d)). Among the DEGs, 114 common genes with upregulation and 308 common genes with downregulation were identified in both the TCGA and GSE106191 databases (Figures 1(e) and 1(f)).

### 3.2. Bioinformatics Analysis of DEGs in EC

**Figure 2:**
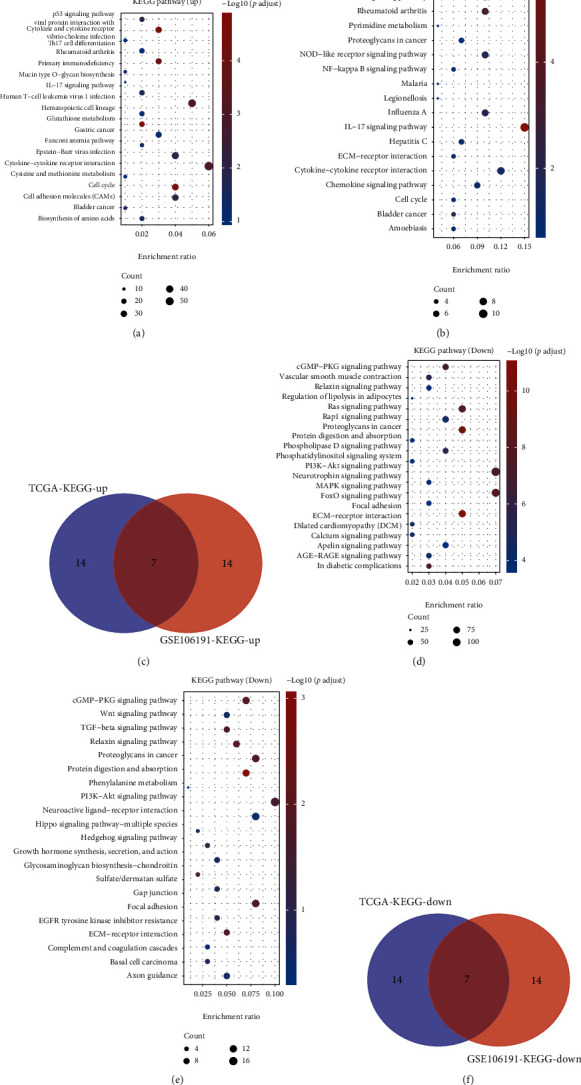
KEGG pathway analysis of DEGs in EC. (a) KEGG pathway analysis of upregulated genes by analyzing the TCGA database. (b) KEGG pathway analysis of upregulated genes by analyzing the GSE106191 database. (c) Venn map analysis of upregulated gene-related pathways. (d) KEGG pathway analysis of downregulated genes by analyzing the TCGA database. (e) KEGG pathway analysis of downregulated genes by analyzing the GSE106191 database. (f) Venn map analysis of downregulated gene-related pathways.

Next, we performed KEGG and GO analyses of DEGs in endometrial carcinoma using TCGA and GSE106191, respectively. As presented in Figure 2, the KEGG analysis showed that the pathways related to DEGs were similar by analyzing either TCGA or GSE106191 (Figures 2(a), 2(b), 2(d), and 2(e)). The KEGG analyses of upregulated genes were related to bladder cancer, cell cycle, cytokine-cytokine receptor interaction, IL17 signaling pathway, cytokine, and cytokine receptor (Figure 2(c)). The KEGG analyses of downregulated genes were related to ECM-receptor interaction, focal adhesion, PI3K-Akt signaling pathway, protein digestion and absorption, proteoglycans in carcinoma, relaxin signaling pathway, and cGMP-PKG signaling pathway (Figure 2(f)).

**Figure 3:**
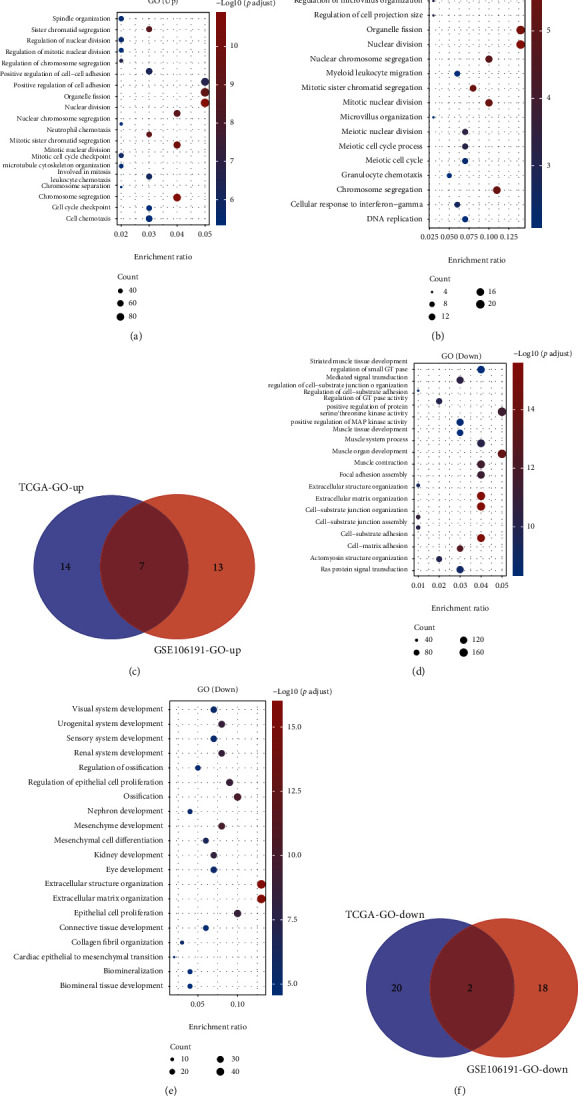
GO analysis of DEGs in EC. (a) GO analysis of upregulated genes by analyzing the TCGA database. (b) GO analysis of upregulated genes by analyzing the GSE106191 database. (c) Venn map analysis of upregulated gene-related pathways. (d) GO analysis of downregulated genes by analyzing the TCGA database. (e) GO analysis of downregulated genes by analyzing the GSE106191 database. (f) Venn map analysis of downregulated gene-related pathways.

Furthermore, the GO analysis also showed that the biological processes related to DEGs were similar by analyzing either TCGA or GSE106191 (Figures 3(a), 3(b), 3(d), and 3(e)). The GO analyses of upregulated genes were related to chromosome segregation, mitotic nuclear division, mitotic sister chromatid segregation, nuclear chromosome segregation, nuclear division, organelle fission, and sister chromatid segregation (Figure 3(c)). The GO analysis of downregulated genes exhibited a relationship to extracellular matrix organization and structure organization (Figure 3(f)).

### 3.3. Identification of Prognosis-Related DEGs in EC

**Figure 4:**
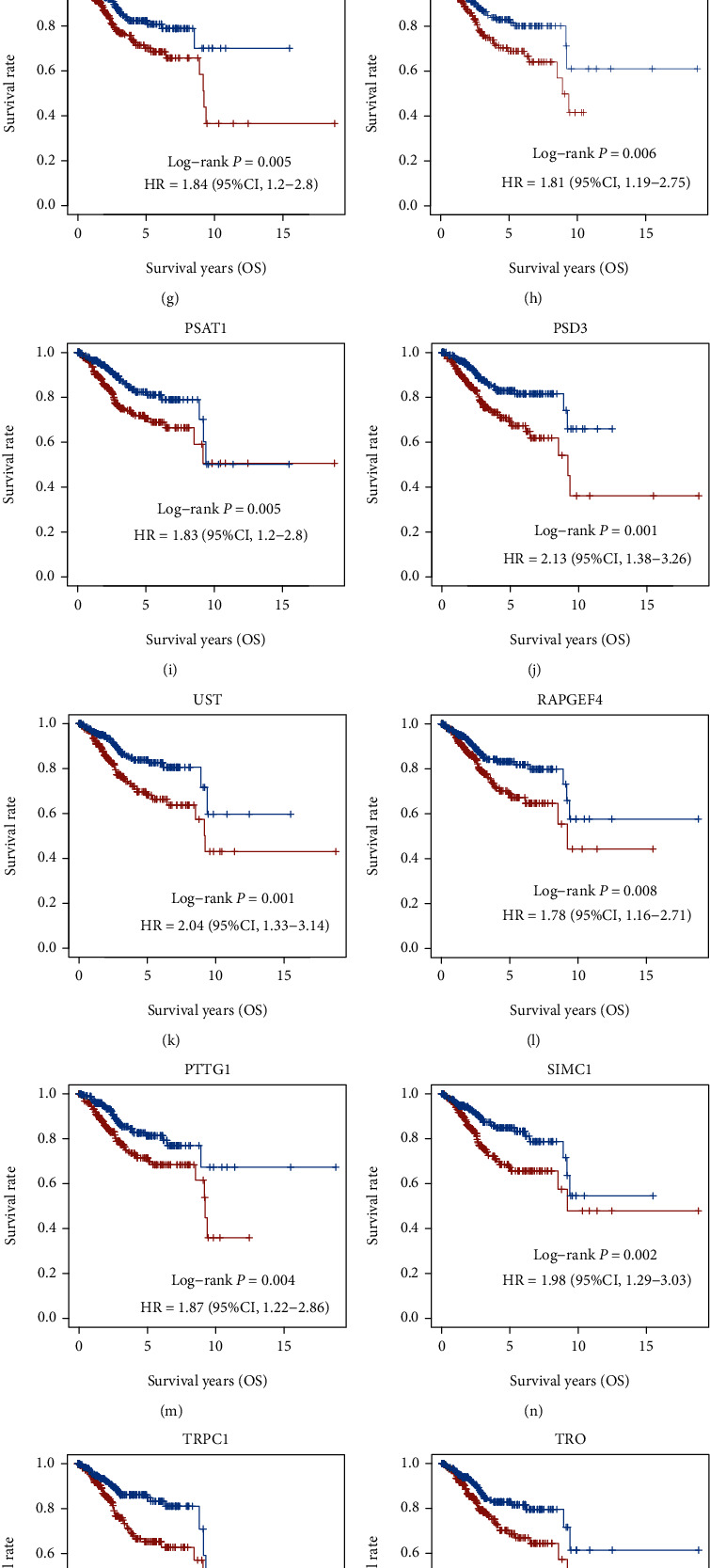
Identification of DEGs related to poor prognosis in EC. (a–s) Among these genes, higher expressions of BEX4, ERFE, FBXO17, GLDC, MAL2, MCM10, METTL7B, OAS1, POLQ, PSAT1, PSD3, PTTG1, RAPGEF4, SIMC1, SRPX, TIMP3, TRO, TRPC1, and UST were correlated to shorter OS time in patients with EC.

**Figure 5:**
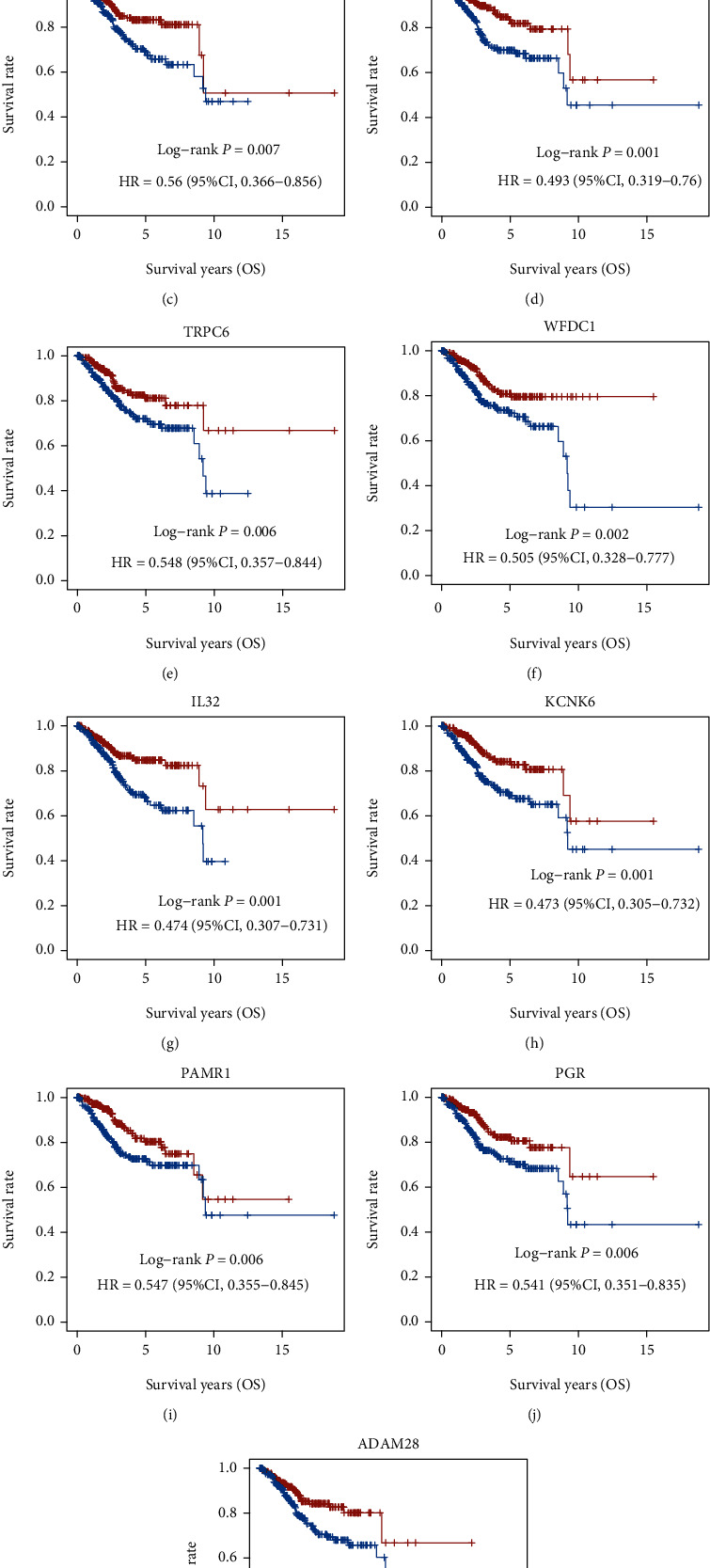
Identification of DEGs related to good prognosis in EC. (a–k) Higher expressions of WFS1, GREB1, FAM189A2, ANKRD22, WFDC1, TRPC6, KCNK6, IL32, PGR, PAMR1, and ADAM28 were correlated to longer OS time in patients with EC.

In the above analysis, we identified 572 DEGs in EC. In order to identify prognosis-related DEGs in ECs, we carried out the Kaplan-Meier Plotter to determine the correlation of the DEG expression with the overall survival (OS) time in EC. Finally, we identified 30 DEGs that were related to the prognosis of EC, including KCNK6, IL32, FAM189A2, WFS1, GREB1, WFDC1, PGR, PAMR1, TRPC6, ADAM28, ANKRD22, GLDC, RAPGEF4, MCM10, TRO, OAS1, BEX4, PSAT1, METTL7B, TIMP3, FBXO17, PTTG1, POLQ, MAL2, SIMC1, ERFE, TRPC1, SRPX, UST, and PSD3. Among these genes, higher expressions of BEX4, ERFE, FBXO17, GLDC, MAL2, MCM10, METTL7B, OAS1, POLQ, PSAT1, PSD3, PTTG1, RAPGEF4, SIMC1, SRPX, TIMP3, TRO, TRPC1, and UST were correlated to shorter OS time in patients with EC (Figures 4(a)–4(s)). However, higher expressions of WFS1, GREB1, FAM189A2, ANKRD22, WFDC1, TRPC6, KCNK6, IL32, PGR, PAMR1, and ADAM28 were correlated to longer OS time in patients with EC (Figures 5(a)–5(k)).

### 3.4. Establishing a Prognostic Gene Model in the TCGA Cohort

**Figure 6:**
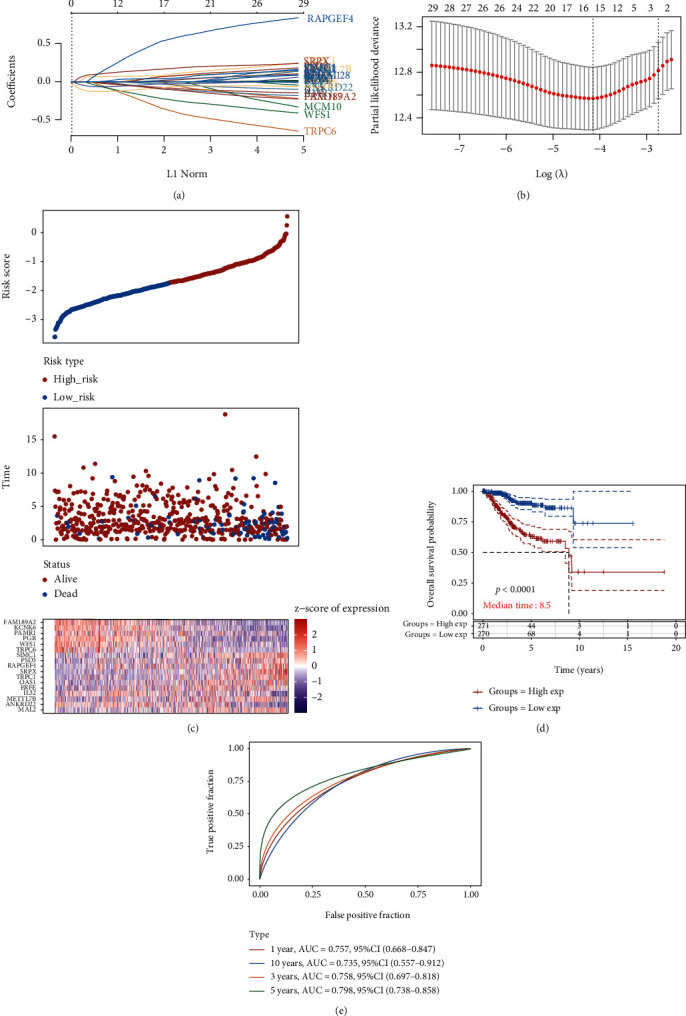
Establishing a prognostic gene model in the TCGA cohort. (a) LASSO coefficients profiles of 30 prognostic genes in EC. (b) LASSO regression with tenfold cross-validation obtained 17 prognostic genes using minimum lambda value. (c) 543 patients with EC were divided equally into low-risk group and high-risk group on the basis of the median score calculated by the risk score formula. (d) Lower OS time was observed in the high-risk group of EC patients, in comparison with the low-risk group of EC patients by Kaplan-Meier Plotter analysis. (e) Time-dependent receiver operating characteristic (ROC) analysis to assess the sensitivity and specificity of the prognostic model.

We utilized the least absolute shrinkage and selection operator (LASSO) Cox regression analysis to establish the prognostic gene model. Figures 6(a) and 6(b) revealed a 17-gene signature constructed in the light of the optimum *λ* value. We calculated the risk score as follows: risk score = (4*e* − 04)∗MAL2 + (−0.0263)∗ANKRD22 + (0.0493)∗METTL7B + (0.0688)∗IL32 + (0.0022)∗ERFE + (0.01)∗OAS1 + (0.0745)∗TRPC1 + (0.1564)∗SRPX + (0.4778)∗RAPGEF4 + (0.0496)∗PSD3 + (0.0383)∗SIMC1 + (−0.3016)∗TRPC6 + (−0.2001)∗WFS1 + (−0.0341)∗PGR + (−0.0821)∗PAMR1 + (−0.0912)∗KCNK6 + (−0.0813)∗FAM189A2. 543 patients with EC were divided equally into the low-risk group and the high-risk group on the basis of the median score calculated by the risk score formula. Compared to patients in the low-risk group, those in the high-risk group displayed a larger death toll and a shorter survival time (Figure 6(c)). Our data revealed that an obviously lower OS time was observed in the high-risk group of EC patients, in comparison with the low-risk group of EC patients by Kaplan-Meier Plotter analysis (Figure 6(d)). We applied time-dependent receiver operating characteristic (ROC) analysis to assess the sensitivity and specificity of the prognostic model. And our results indicated that the area under the ROC curve (AUC) was 0.757 for 1-year, 0.758 for 3-year, 0.798 for 5-year, and 0.735 for 10-year survival (Figure 6(e)).

### 3.5. Genetic Alteration Differences of Prognostic Genes in EC Patients

**Figure 7:**
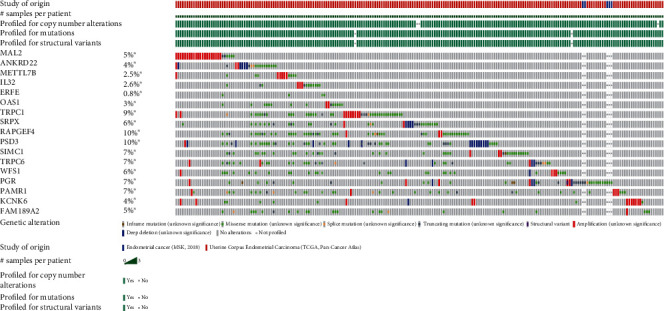
Genetic alteration differences of prognostic genes in EC patients. The mutation rates of prognostic genes for EC ranged from 0.8% to 10% for individual genes (MAL2, 5%; ANKRD22, 4%; METTL7B, 2.5%; IL32, 2.6%; ERFE, 0.8%; OAS1, 3%; TRPC1, 9%; SRPX, 6%; RAPGEF4, 10%; PSD3, 10%; SIMC1, 7%; TRPC6, 7%; WFS1, 6%; PGR, 7%; PAMR1, 7%; KCNK6, 4%; and FAM189A2, 5%).

Furthermore, genetic alteration of prognostic genes in EC was analyzed using the cBioPortal database, which included 726 patients from seven related studies. We observed that the mutation rates of prognostic genes for EC ranged from 0.8% to 10% for individual genes (MAL2, 5%; ANKRD22, 4%; METTL7B, 2.5%; IL32, 2.6%; ERFE, 0.8%; OAS1, 3%; TRPC1, 9%; SRPX, 6%; RAPGEF4, 10%; PSD3, 10%; SIMC1, 7%; TRPC6, 7%; WFS1, 6%; PGR, 7%; PAMR1, 7%; KCNK6, 4%; and FAM189A2, 5%). Among these genes, RAPGEF4 and PSD3 were found to have the highest mutation rate in EC, which are mutated in about 10% EC cases (Figure 7).

### 3.6. Validation of 17 Prognosis-Related Hub Gene Expressions in EC

**Figure 8:**
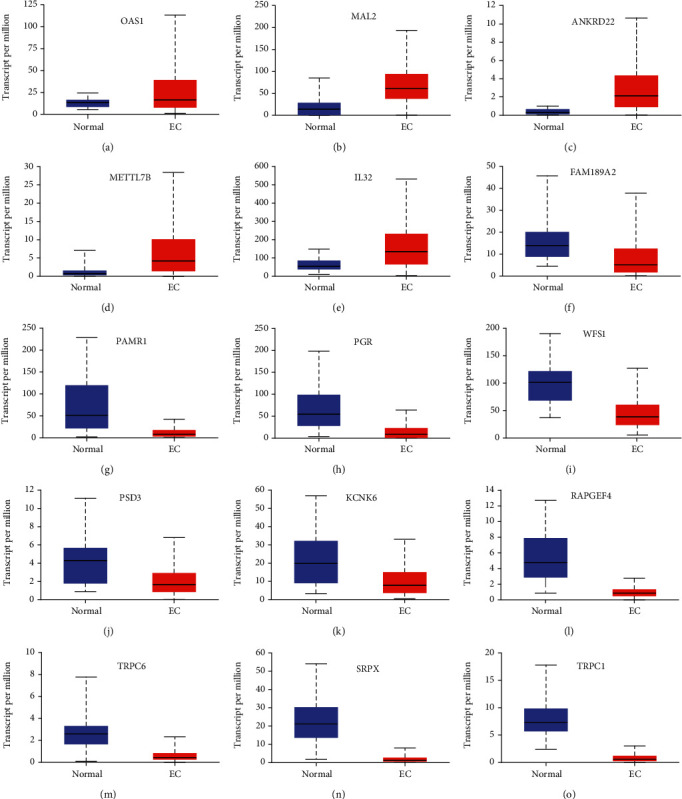
Validation of 17 prognosis-related hub gene expressions in EC.

For verification of the bioinformatics analysis data in-depth, UALCAN databases were used to confirm our findings. As presented in Figure 7, compared to normal samples, OAS1, MAL2, ANKRD22, METTL7B, and IL32 were dramatically upregulated in EC samples, whereas TRPC1, SRPX, RAPGEF4, PSD3, SIMC1, TRPC6, WFS1, PGR, PAMR1, KCNK6, and FAM189A2 were greatly downregulated in EC samples (Figures 8(a)–8(o)).

**Figure 9:**
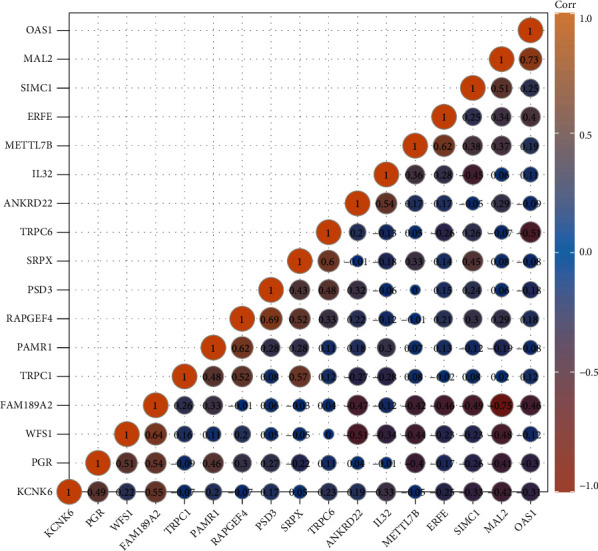
17 prognosis-related hub gene expressions were correlated to each other in EC. The expressions of 17 prognosis-related hub genes were significantly correlated based on Pearson correlation.

We also analyzed the correlation among these 17 prognosis-related hub genes in EC. Our results showed that the expressions of the 17 prognosis-related hub genes were significantly correlated based on Pearson correlation. The most significantly negatively correlated gene pairs included FAM189A2-MAL2, MAL2-FAM189A2, TRPC6-OAS1, OAS1-TRPC6, WFS1-ANKRD22, and ANKRD22-WFS1. And the most significantly positively correlated gene pairs included SIMC1-MAL2, MAL2-SIMC1, PGR-WFS1, WFS1-PGR, RAPGEF4-TRPC1, TRPC1-RAPGEF4, RAPGEF4-SRPX, SRPX-RAPGEF4, IL32-ANKRD22, ANKRD22-IL32, FAM189A2-PGR, PGR-FAM189A2, FAM189A2-KCNK6, KCNK6-FAM189A2, SRPX-TRPC1, TRPC1-SRPX, TRPC6-SRPX, SRPX-TRPC6, PAMR1-RAPGEF4, RAPGEF4-PAMR1, ERFE-METTL7B, METTL7B-ERFE, FAM189A2-WFS1, WFS1-FAM189A2, PSD3-RAPGEF4, RAPGEF4-PSD3, OAS1-MAL2, and MAL2-OAS1 (Figure 9).

## 4. Discussion

Emerging studies revealed that the occurrence of EC resulted from the abnormally expressed multiple carcinoma-related genes [29, 30], amid which have been shown to display a relationship to EC's susceptibility and progression [29, 30]. Many molecular biology methods have been used to identify biomarkers of cancers [31–34]. Nevertheless, the majority of them merely concentrated on a single genetic factor, limiting these biomarkers' reliability.

Our current study discovered more EC-related genes with differential expression than previous researches, indicating they may play importantly in the mechanism of EC. Our data revealed 114 common genes with upregulation and 308 common genes with downregulation in EC samples in comparison with normal samples. Bioinformatics analysis showed that these genes were significantly correlated to multiple key signaling in EC, such as the cGMP-PKG signaling pathway. Furthermore, we identified that 30 DEGs were related to the prognosis of EC, comprising KCNK6, IL32, FAM189A2, WFS1, GREB1, WFDC1, PGR, PAMR1, TRPC6, ADAM28, ANKRD22, GLDC, RAPGEF4, MCM10, TRO, OAS1, BEX4, PSAT1, METTL7B, TIMP3, FBXO17, PTTG1, POLQ, MAL2, SIMC1, ERFE, TRPC1, SRPX, UST, and PSD3.

Here, we conducted GO and KEGG analyses of the involved biological processes and pathways related to these DEGs in EC's progression. The pathway analysis of these DEGs showed that the interconnected network of genes participated in the cyclic guanosine monophosphate- (cGMP-) protein kinase G (PKG) signaling pathway. As previously described, the contractility of the uterine smooth muscle is of importance for the periodic shedding of the endometrial lining and the expulsion of the fetus during parturition. There was one study showing that the nitric oxide- (NO-) cGMP signaling pathway participates in the relaxation of the smooth muscle. cGMP-dependent PKG, which is essential for reducing cytoplasmic calcium and muscle tension, was the downstream target of the NO-cGMP pathway [35]. PKG was responsible for controlling the uterine smooth muscle tone which produced force near menstruation and regulated blood flow to the endometrial lining. The above data together confirmed that PKG functioned crucially in controlling the contraction of the uterine and vascular smooth muscle during the periodical menstruation [35]. PI3K-AKT signaling is one of the most important pathways in our study. PI3K-AKT signaling could be antagonized by the tumor suppressor phosphatase and tensin homolog (PTEN) which was reported to be usually mutated in several sorts of neoplasms, such as the endometrium, skin, brain, and prostate cancers [36–38]. PTEN has a powerful phosphatase activity, which is the best characterized physiological function leading to the tumor suppressor function of PTEN.

The IL17 signaling pathway was also one of the important pathways detected here. In inflammatory mediators, more and more evidence emphasizes the role of the interleukin-17 (IL17) cytokine family in malignant diseases. IL17 is becoming a crucial cytokine to promote and develop carcinomas by maintaining a chronic inflammatory microenvironment which is conducive to tumor formation [39, 40]. While IL17 may regulate chemokines and cytokines in gynecologic cancers, Toll-like receptors may function importantly in the gynecologic carcinomas' development via triggering an inflammatory response and cell survival in the microenvironment of the tumor [41].

Our study generated a signature featuring17 prognosis-related genes (MAL2, ANKRD22, METTL7B, IL32, ERFE, OAS1, TRPC1, SRPX, RAPGEF4, PSD3, SIMC1, TRPC6, WFS1, PGR, PAMR1, KCNK6, and FAM189A2) and demonstrated that they were utilized as predictors of OS in EC patients. We obtained many genes that were previously reported to be involved in endometriosis patients or endometrial stromal cells. For instance, the members of the transient receptor potential (TRP) ion channel superfamily, known as having the calcium permeability, has become pivotal modulators in the endometrium. Previous studies have shown that TRPC1 and TRPC6 were highly expressed in the entire endometrium during the periodical menstruation. Additionally, TRPV2, TRPV4, TRPC1/4, and TRPC6 were found in human endometrial stromal cells (hESCs) from patients with endometriosis [25]. Previous reports suggested that the cAMP2-activated exchange protein (EPAC2, RAPGEF4), another cAMP mediator, took part in endometrial stromal cell differentiation via regulating calreticulin (CALR) expression [42]. Compared with the control group, the level of interleukin-32 (IL32) in peritoneal fluid (PF) in women with endometriosis was significantly higher. The endometrial cells treated with IL32 in vitro significantly enhanced cell viability, proliferation, and invasion capabilities [43]. In silico methods can distinguish many key genes related to the maintenance of telomeres, which were unknown to the occurrence and prognosis of EC before, including WFS1. Prognostic biomarkers of EC are essential for ameliorating risk assessment before and after surgery and making guided- treatment decisions. PGR and PTEN were one of the most clinically valuable EC prognostic biomarkers. In our research, we showed that significant genes with upregulation in EC samples included OAS1, MAL2, ANKRD22, METTL7B, and IL32 after comparison with normal samples. Obvious genes with downregulation in EC samples comprised TRPC1, SRPX, RAPGEF4, PSD3, SIMC1, TRPC6, WFS1, PGR, PAMR1, KCNK6, and FAM189A2. And correlation analysis showed that our results showed that the expressions of 17 prognosis-related hub genes were significantly correlated based on Pearson correlation.

However, there are still several limitations in our literature. Firstly, the number of samples is limited, which should be enlarged in the following study, and all the samples are from public datasets; our own data is also very important. Secondly, we need to conduct more researches to expound the function and potential mechanisms of these promising biomarkers in the progression of EC.

## 5. Conclusion

In summary, our findings revealed 114 common genes with upregulation and 308 common genes with downregulation in EC samples relative to normal ones. Bioinformatics analysis showed these genes exhibited a significant relationship to multiple signaling and biological processes, such as the cGMP-PKG signaling pathway and PI3K-AKT signaling. Moreover, we constructed a 17-gene signature to make a prediction of OS in EC patients using the TCGA cohorts. We collectively supplied a potential gene signature for the prediction of EC patients' prognosis.

## Data Availability

All the data and material were presented in GSE106191 and TCGA (https://portal.gdc.cancer.gov/).
